# On the occurrence, origin, and intake of the nuclides, ^210^Po and ^210^Pb, in sclerotia of *Wolfiporia cocos* collected in China

**DOI:** 10.1007/s11356-021-18313-5

**Published:** 2022-01-03

**Authors:** Dagmara Strumińska-Parulska, Jerzy Falandysz, Aleksandra Moniakowska

**Affiliations:** 1 Toxicology and Radiation Protection Laboratory, Faculty of Chemistry, University of Gdańsk, 80-308 Gdańsk, Poland; 2 Department of Toxicology, Faculty of Pharmacy, Medical University of Lodz, 1 Muszyńskiego Street, 90-151 Lódź, Poland; 3 Medicinal Plants Research Institute, Yunnan Academy of Agricultural Sciences, Kunming, 650200 China

**Keywords:** Radioactivity, ^210^Po and ^210^Pb, Foods, Food toxicology, Mushrooms, Nutrition supplements, Alternative medicine products

## Abstract

The dried sclerotium of the fungus *Wolfiporia cocos* is edible and has medicinal value. This study aimed to understand the accumulation of radioactivity arising from the alpha ^210^Po, and beta-emitting ^210^Pb, in the sclerotium’s shell and core and assess a potential effective dose for consumers. Sclerotia were collected in the wild and from cultivars in China’s Anhui and Yunnan provinces. The mean values of ^210^Po activity concentration levels were 0.36 Bq kg^−1^ dry weight in the core and 12.0 Bq kg^−1^ dw in the shell; ^210^Pb activities were 0.43 and 9.84 Bq kg^−1^ dw, respectively. The potential effective radiation doses from core layers (as a major raw material of the sclerotium) ranged from 0.13 to 3.43 µSv kg^−1^ dw from ^210^Po decay and from 0.11 to 1.52 µSv kg^−1^ dw from ^210^Pb decay. Corresponding values for shell ranged from 0.80 to 42.4 for ^210^Po and from 0.53 to 13.6 µSv kg^−1^ dw for ^210^Pb. In general, the intake of *W. cocos* sclerotia varies between consumers, but this would not significantly change the effective radiation doses from ^210^Po and ^210^Pb isotopes. The consumption thus appears to be safe from a radiological protection point of view.

**Graphical abstract:**

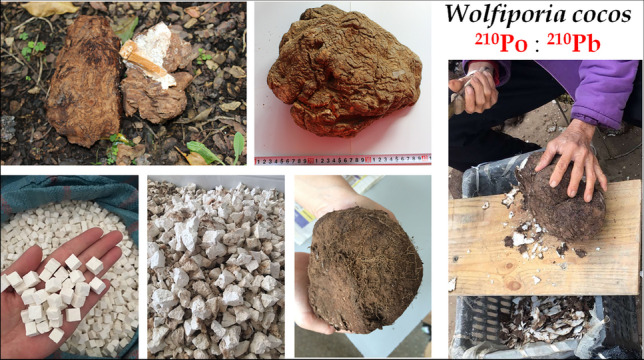

## Introduction

*Wolfiporia cocos* (Schwein.) Ryvarden *et* Gilb. is one of the earliest and most commonly utilized fungus in traditional China medicine as well as in Japan (Leung and Foster, 1996; Ríos, 2011; Kobira et al. 2012; Wang et al. 2013). It is also a part of the complementary economic activity of the rural regions of the Yunnan province in China, e.g., annual export in the years 2011–2016 averaged 9,279.73 t (Chi et al., 2018). Like almost any macroscopic fungus (macromycete, mushroom), this species has several other Latin names, e.g., *Sclerotium cocos, Poria cocos*, *Wolfiporia extensa*, *Daedalea extensa*, *Macrohyporia extensa*, *Macrohyporia cocos*, *Pachyma cocos*, or poria (Index Fungorum 2020). In present-day China, it is known as “fu-ling” or “hoelen,” including the bark “fu-ling-pi,” an outermost reddish layer “chih-fu-ling,” the middle white layer “bai-fu-ling,” and the core “fu-shen” (Leung and Foster, 1996; Ríos, 2011). It is a wood-decaying fungus from the Polyporaceae family that in nature grows underground on pine tree (*Pinus yunnanensis* Franch.) roots. When domesticated, it grows on dead and buried pine wood (Wang et al., 2013). For the saprophytic *W. cocos,* the wooden substrate and, indirectly, the soil solution are sources of minerals and trace elements. Its sclerotium has a large coconut-shaped, dense mycelial formation buried underground (in wood or soil) which usually weighs less than a kilogram. Occasionally, it can reach up to several kilograms in the wild (Falandysz et al., 2021).

The moisture content of the freshly harvested sclerotium of *W. cocos* is 50%, and when cleaned and chopped, it is traditionally air-dried in the shade (Kubo et al., 2006). About 10% of the medicinal preparations listed in the *Pharmacopoeia of the People’s Republic of China* (2000) contain this sclerotium (Leung and Foster, 1996; Song et al., 2002; Ríos 2011). The primary constituent of the internal part is fiber in the form of beta-glucan and constitutes up to 98% of the dried fungal mass, and there are barely any lipids (< 0.15%) or protein (1%) in the fungus (Yang et al., 2014). Polysaccharides show strong antitumor and immunomodulatory effects (Ríos 2011). Also, its low molecular weight tetracyclic triterpenes have been found to have immunostimulating, antiviral, tumor inhibitory, and cytotoxic properties (Wang et al., 2013; Nie et al., 2020; Lu et al., 2021). In folk medicine, the *W. cocos* sclerotium is prescribed for diarrhea, dampness (mucus) in the spleen (according to traditional Chinese medicine), and insomnia, as a sedative to calm the mind and refresh the spirit (Wong and Cheung, 2008). It is also used as an additive in a snack (Wang et al., 2015a, b). According to Ríos (2011), the recommended daily dosages of sclerotium for reinforcing the spleen and stomach are 9 to 18 g (dry weight, dw) or 30 to 45 g for edema and 3 to 9 g for sedation or the treatment of palpitations. The *Chinese Compendium of Materia Medica* does not give a precise daily dosage of sclerotium (it shows a number of approximate dosages that make it difficult to calculate a typical daily dosage). In the *2015 Pharmacopoeia of the People’s Republic of China*, the recommended daily intakes of sclerotium were 10–15 g and 15–30 g dw for the core and shell (outer part), respectively.

In Yunnan, the karstic landscape with high regional heterogeneity, carbonate rocks, and karst forests, rich in plant biodiversity and dominated by evergreen and deciduous broadleaf trees, covers a large provincial region from the east and west, with a non-karst region in the center. At the same time, soils there are largely lateritic red earths, with some areas containing red and yellow earths and latosols, which are considered polymetallic (locally/regionally enriched, e.g., in Ag, As, Cd, Co, Cu, Cr, Fe_2_O_3_, Hg, Mo, Ni, Pb, Sn, V or W) with baseline values that are governed by the important influence of the weathering of the parent rocks (Cheng et al. 2021; Falandysz et al. 2015a, b; He et al. 2004; Li and Walker, 1986; Liang et al. 2021; Liu et al., 2020a, b; Liu et al., 2020a; Wang et al. 2019a; Wang et al. 2019b; Wang et al. 2021). Yunnan province is one of the regions connected to the uranium and coal mining industry (Ren et al., 1999; Li et al., 2006; Wang et al., 2015a; Chen et al., 2017). Some coals in Yunnan are also classified as coal-hosted uranium deposits (Chen et al., 2017). The mineral composition of the soil is an essential factor determining root and mycelial uptake and the occurrence of some minerals and trace elements in aboveground green vegetation and mushrooms in Yunnan (Cheng et al. 2021; Falandysz et al. 2015c, 2017a, 2020a; Komorowicz et al. 2019; Wang et al. 2019b; Zhang et al. 2019, 2020; Zou et al. 2018).

To our knowledge, the results available on the mineral composition and inorganic contaminant content in the sclerotia of *W. cocos* and its processed products are limited (Wiejak et al. 2016; Sun et al. 2016; 2017; Falandysz et al. 2017b and 2020b). The information available on the radionuclides content in *W. cocos* is even scarcer. So far, only the results on the contents of three gamma-emitting radioisotopes, namely ^134^Cs, ^137^Cs, and ^40^ K, are available (Falandysz et al. 2021; Wang et al. 1998; Wang et al. 2015a, b). Such factual data are important for consumers of *W. cocos* due to its widespread use in traditional medicine. Nowadays, *W. cocos* has become more popular in other parts of the world. It is available in many retail and online shops under the trade name “poria” or “fu ling” and is recommended as a dietary supplement.

The present study aimed to investigate the occurrence and spatial variability of two radionuclides, ^210^Po and ^210^Pb, in shell and core parts of *W. cocos* sclerotia collected across Yunnan. These radionuclides occur naturally in the environment as uranium daughters and are known to significantly contribute to the radiation dose of every population. There are two primary sources of ^210^Po and ^210^Pb isotopes for humans: foods and aerosols, and ^210^Po, in particular, is one of the most critical natural radionuclides to which people are exposed (Carvalho, 1995). ^210^Po and ^210^Pb, together with radium (^226^Ra) and potassium (^40^ K), deliver the highest natural dose to living organisms from natural radiation sources (Bem, 2005). If radon emanates, the higher the natural radiation background, the higher the doses from ingested ^210^Po and ^210^Pb decay. Therefore, research has focused on knowing the spatial occurrence distribution, assessing the potential intake, and evaluating the hypothetical effective exposure doses from the ^210^Po and ^210^Pb contained in *W. cocos* nutrition products that could be received by individuals and for which scientific data are not available.

## Materials and methods

### Collection and initial preparation

Sclerotia of *Wolfiporia cocos* (Schwein.) Ryvarden *et* Gilb. were collected from 26 locations across Yunnan and one from the Anhui province (Table 1). The locations were randomly selected at altitudes between 617 and 2578 m above sea level (Table 1). No specific documents were required for the defined field studies as no protected species were sampled. Sclerotia of wild-growing and cultivated *W. cocos* were collected (4–63 individuals *per* location). The white inner part (core) of the sclerotia and the brown external part (shell) were separated for the study. In samples 1–9, only the internal parts were available for radiochemical analysis. Six subsamples were taken (each ca. 300 g) and pooled to make a bulk sample. The sclerotial parts were cut into small pieces, dried at 65 °C, and powdered using a porcelain mortar. The dried samples were stored in new sealable polythene bags under dry and clean conditions in a herbal materials depository room before further analyses (Falandysz et al., 2020a).

**Table 1:** Characteristic of *W. cocos* samples; *specimens from cultivation

No	Collection site	Altitude (m)	Latitude	Longitude	No of individuals
1	Yuanjiang, Yuxi	1588	N23° 48′23.21″	E102° 51′59.41″	6
2	Jianshui, Honghe	1960	N23° 59′10.14″	E103° 47′29.55″	6
3	Tengchong, Baoshan	1512	N24° 41′07.80″	E98° 36′52.60″	6
4	Huize, Qujing	2578	N26° 28′42.23″	E103° 13′19.90″	6
5	Shiping, Honghe	1462	N23° 46′23.46″	E102° 25′37.57″	6
6	Kaiyuan, Honghe	1628	N24° 00′11.71″	E102° 59′0.15″	6
7	Octagonal town, Chuxiong	2061	N24° 52′38.66″	E100° 56′32.24″	6
8	Xincun town, Chuxiong	2056	N24° 40′48.91″	E101° 16′57.04″	6
9	Nanhua, Chuxiong	2245	N25° 01′47.28″	E100° 51′21.06″	6
10	Yongping, Dali	1943	N25° 32′09.82″	E99° 41′05.70″	20
11	Lanping, Nujiang	2495	N26° 39′48.19″	E99° 11′23.84″	22
12	Hongta, Yuxi*	1720	N24° 25′54.7″	E102° 31′5.6″	21
13	Changning, Baoshan*	2011	N24° 28′08.51″	E99° 30′11.47″	22
14	Zhenyuan, Pu'er*	1892	N23° 49′12.04″	E100° 44′26.82″	5
15	Shuangbai, Chuxiong*	2062	N24° 41′28.21″	E101° 38′57.42″	21
16	Baozhu, Wenshan	1504	N23° 28′29.99″	E103° 56′50.35″	63
17	Lanping, Nujiang	2495	N26° 39′48.19″	E99° 11′23.84″	8
18	Ninglang, Lijiang*	2560	N26° 52′59.49″	E100° 55′39.13″	38
19	Tengchong, Baoshan*	1582	N25° 25′20.32″	E98° 39′08.66″	18
20	Shuangjiang, Lincang*	1438	N23° 20′55.478″	E100° 0′17.0856″	15
21	Yunlong, Dali*	2066	N25° 38′9.7512″	E99° 7′55.0811″	13
22	Jinggu, Pu'er*	1077	N23° 25′13.454″	E100° 24′15.678″	19
23	Shuangjiang, Lincang*	1052	N23° 28′40.537″	E99° 50′16.134″	11
24	Mojiang, Pu'er*	1979	N23° 4′3.4824″	E101° 58′35.501″	8
25	Simao, Pu'er*	1474	N22° 44′55.295″	E101° 3′22.6368″	4
26	Yuexi, Anhui Province*	617	N31° 4′8.584″	E116° 6′49.1472″	16

### Radiochemical analysis

Analytical samples of *W. cocos* (~ 5 g) were treated with 9.8 mBq of ^209^Po before radiochemical analysis. All prepared samples were digested using concentrated 65% HNO_3_. After digestion, the residues were dissolved in 0.5 M HCl, ascorbic acid was added, and polonium was autodeposited on a 100% pure silver disc. The activities of ^209^Po and ^210^Po were measured using an alpha spectrometer (Alpha Analyst, Canberra) equipped with semiconductor silicon detectors of a 450 mm^2^ active surface barrier, 18 keV resolution, and 30–35% efficiency, calibrated using a certified solid Isotrak source ^237^Np + ^241^Am + ^244^Cm (Strumińska-Parulska and Olszewski 2018). The ^210^Pb analysis was connected to the polonium analysis—^210^Pb content determination based on its daughter ^210^Po activity measurement. After 6 months, the samples were spiked with 9.8 mBq of ^209^Po again, digested in concentrated 65% HNO_3_, and the activities of the ingrown ^210^Po were measured in the alpha spectrometer. The residue after the first deposition was heated very strongly, and a few ml of hydrogen peroxide were added. Thanks to this, as we already checked, all residual Po tracer was evaporated and removed. All radionuclides activities at the time of the mushroom collection were calculated using the simplified equation for the daughter activity as a function of time (Strumińska-Parulska and Olszewski 2018). The ^210^Po and ^210^Pb yield in the analyzed mushroom samples ranged from 80 to 99%. The results of ^210^Po and ^210^Pb activity concentrations were reported with measurement uncertainty. The accuracy, as well as precision, of the radiochemical method was positively evaluated using IAEA reference materials (IAEA-414), and both estimated at less than 5%. The minimum detectable activity (MDA) was 0.10 mBq. The interpolation maps were prepared using QGIS software (QGIS Development Team).

Statistic tests exposed a positive skewed non-normal distribution of the results, and non-parametric tests, mainly *U*-test (Mann–Whitney), were used. The Spearman correlation coefficient was used for surveying the relationship between chosen variables. To find out the hidden patterns in the dataset and identify the associations between observations and variables, the principal component analysis (PCA) was used. Data were standardized, the analysis was based on the correlation matrix, and a fiducial significance level of *p* < 0.05 was chosen. The Kaiser rule has been used while selecting the number of components, and only factors with eigenvalues considerably greater than 1 were retained. The length of the PCA lines indicates the variance of the variables. The most influential variables on a given principal component are displayed as the most extended vectors. The angles between the vectors reflect their correlations. Small angle between the vectors suggests a strong positive correlation, whereas a large one suggests a weak negative correlation. Points close together in the two principal component space poses similar scores, i.e., have similar values of the original variables.

## Results and discussion

### ^*210*^*Po and *^*210*^*Pb activity concentrations distribution in W. cocos*

**Fig. 1:**
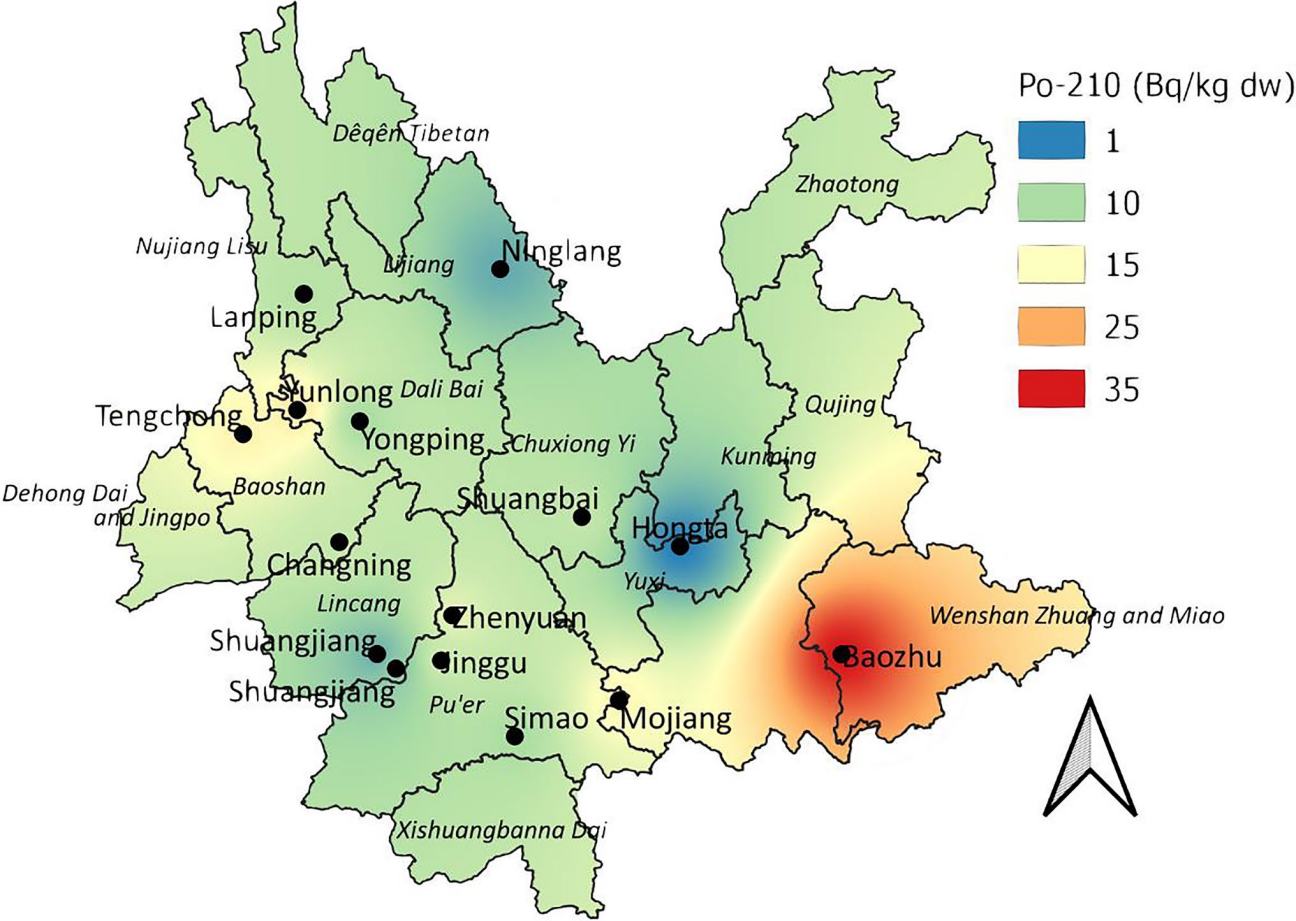
Interpolation for ^210^Po activity concentrations in the external part of *W. cocos* collected in Yunnan province

The study results on ^210^Po activity concentrations in the sclerotia samples of *W. cocos* collected in Yunnan are given in Table 2. The core’s median and mean value of ^210^Po activity concentrations were calculated at 0.20 and 0.36 Bq kg^−1^ dw, with 11.5 and 12.0 Bq kg^−1^ dw in the shell, respectively. Both maximum and minimum ^210^Po activities were measured in the samples. The results indicate noticeable differences between ^210^Po activities in the internal and external parts of all analyzed pools of sclerotia. The median and mean value of the ^210^Po activities ratio of the external part to the internal part has been calculated at 41.0 and 40.4 Bq kg^−1^ dw. The maximum ^210^Po activity in the internal parts of *W. cocos* was seen in the samples from the Baozhu area (Wenshan Prefecture) (2.86 Bq kg^−1^ dry weight), and the lowest activity was noted in samples from Jianshui (Honghe) (0.11 Bq kg^−1^ dw). Among the determined ^210^Po activities in the external parts of *W. cocos* sclerotia, the maximum value was observed in samples from Baozhu (Wenshan) (35.3 Bq kg^−1^ dw), while the sample with the lowest measured value was collected in Hongta (Yuxi) (0.67 Bq kg^−1^ dw) (Table 2). The study results on ^210^Po activities in external parts of analyzed mushrooms sclerotia are also visualized as an interpolation map, showing that ^210^Po activities were relatively elevated in the south-eastern part of Yunnan (Fig. 1).

**Table 2:** ^210^Po and ^210^Pb average values and measurement uncertainty of activity concentrations in the sclerotia of *W. cocos*; *cultivated mushrooms

No	Collection site	^210^Po (Bq kg^−1^ dw)		^210^Pb (Bq kg^−1^ dw)	
		Internal part	External part	Internal part	External part
1	Yuanjiang, Yuxi	0.12 ± 0.02	n/a	0.24 ± 0.02	n/a
2	Jianshui, Honghe	0.11 ± 0.01	n/a	0.20 ± 0.02	n/a
3	Tengchong, Baoshan	0.13 ± 0.01	n/a	0.17 ± 0.02	n/a
4	Huize, Qujing	0.14 ± 0.01	n/a	0.26 ± 0.03	n/a
5	Shiping, Honghe	0.12 ± 0.01	n/a	0.17 ± 0.02	n/a
6	Kaiyuan, Honghe	0.13 ± 0.02	n/a	0.21 ± 0.02	n/a
7	Octagonal town, Chuxiong	0.65 ± 0.04	n/a	0.77 ± 0.04	n/a
8	Xincun town, Chuxiong	0.16 ± 0.02	n/a	0.28 ± 0.02	n/a
9	Nanhua, Chuxiong	0.33 ± 0.02	n/a	0.34 ± 0.03	n/a
10	Yongping, Dali	0.17 ± 0.01	9.96 ± 0.33	0.24 ± 0.02	9.60 ± 0.65
11	Lanping, Nujiang	0.45 ± 0.04	8.35 ± 0.30	0.67 ± 0.05	8.62 ± 0.55
12	Hongta, Yuxi*	0.13 ± 0.02	0.67 ± 0.05	0.16 ± 0.02	0.77 ± 0.06
13	Changning, Baoshan*	0.66 ± 0.05	11.7 ± 0.83	1.05 ± 0.06	10.3 ± 0.70
14	Zhenyuan, Pu'er*	0.19 ± 0.02	13.7 ± 0.91	0.31 ± 0.02	11.2 ± 0.68
15	Shuangbai, Chuxiong*	0.23 ± 0.02	11.5 ± 0.35	0.27 ± 0.02	10.3 ± 0.38
16	Baozhu, Wenshan	2.86 ± 0.15	35.3 ± 3.00	2.20 ± 0.08	19.7 ± 1.38
17	Lanping, Nujiang	0.73 ± 0.05	13.3 ± 0.36	0.84 ± 0.05	9.71 ± 0.34
18	Ninglang, Lijiang*	0.21 ± 0.02	5.13 ± 0.25	0.27 ± 0.02	4.60 ± 0.21
19	Tengchong, Baoshan*	0.31 ± 0.02	16.4 ± 0.86	0.37 ± 0.03	14.7 ± 0.42
20	Shuangjiang, Lincang*	0.20 ± 0.02	8.23 ± 0.71	0.37 ± 0.03	6.01 ± 0.31
21	Yunlong, Dali*	0.20 ± 0.02	17.5 ± 1.03	0.24 ± 0.02	15.3 ± 0.85
22	Jinggu, Pu'er*	0.30 ± 0.02	12.2 ± 0.63	0.37 ± 0.02	12.1 ± 0.75
23	Shuangjiang, Lincang*	0.14 ± 0.01	5.61 ± 0.58	0.26 ± 0.02	4.93 ± 0.26
24	Mojiang, Pu'er*	0.30 ± 0.03	15.8 ± 0.76	0.38 ± 0.03	11.4 ± 0.37
25	Simao, Pu'er*	0.15 ± 0.02	10.1 ± 0.67	0.20 ± 0.01	9.22 ± 0.59
26	Yuexi, Anhui Province*	0.30 ± 0.03	7.86 ± 0.71	0.36 ± 0.03	8.90 ± 0.56
	Mean value	0.36	12.0	0.43	9.84
	Median value	0.20	11.5	0.28	9.71

*n/a* samples not available

**Fig. 2:**
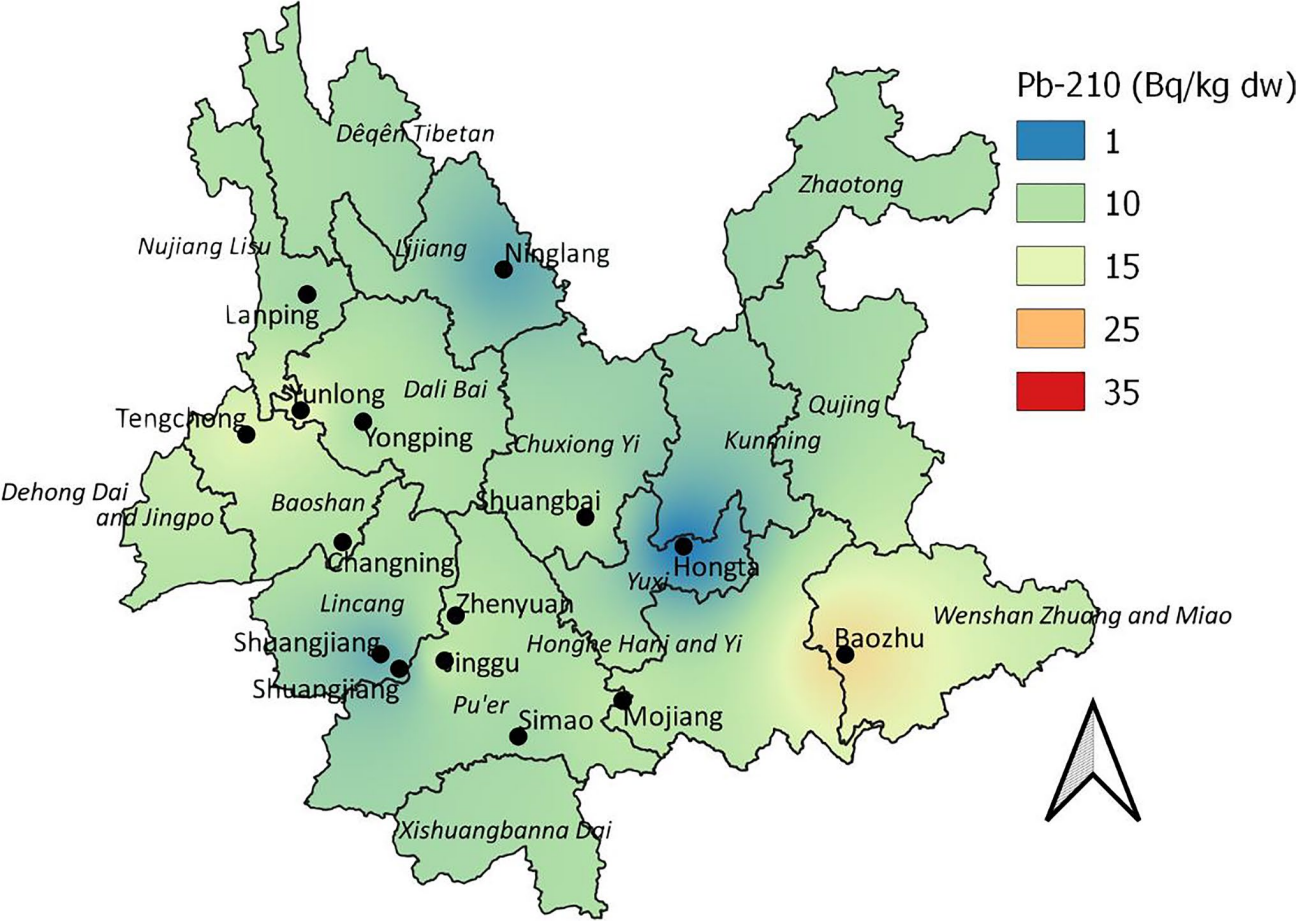
Interpolation for ^210^Pb activity concentrations in the external part of *W. cocos* collected in Yunnan province

The results of ^210^Pb activity concentrations determination in samples of *W. cocos* sclerotia are given in Table 2. The median and mean value of ^210^Pb activity concentrations in the internal part was calculated at 0.28 and 0.43 Bq kg^−1^ dw, while the median and mean value of ^210^Pb activity concentrations in the external part was 9.71 and 9.84 Bq kg^−1^ dw. The obtained results indicated noticeable differences between the activities in the internal and external parts of the sclerotia. The median and mean values of the ^210^Pb activities ratio for the external part relative to the internal were 24.7 and 26.5 Bq kg^−1^ dw. The maximum ^210^Pb activity in the internal parts of sclerotia was observed in samples from Baozhu (Wenshan) (2.20 Bq kg^−1^ dw), and the minimum was seen in a sample from Hongta (Yuxi) (0.16 Bq kg^−1^ dw). The maximum and minimum values in the external part were also seen in the samples from Baozhu (Wenshan) (19.7 Bq kg^−1^ dw) and Hongta (Yuxi) (0.77 Bq kg^−1^ dw), respectively (Table 2). The study results on ^210^Pb activities in external parts of sclerotia are also visualized in a map (Fig. 2), with maximal values being observed in the south-eastern part of Yunnan.

### Statistical analysis and results discussion

Further statistical analysis of ^210^Po and ^210^Pb activity concentrations in sclerotia shows a lack of statistically significant differences in both ^210^Po and ^210^Pb activities between cultivated and wild products (U-test *p*-values 0.49 and 0.73 respectively for external parts and 0.49 and 0.70 for the respective inner parts). There has also been no correlation (Spearman) noted between sampling site altitudes above sea level (h) and ^210^Po and ^210^Pb activities (*r* values from − 0.009 to 0.050). In the studied areas, the content of ^210^Po and ^210^Pb in *W. cocos* samples appears to relate to the amount in the soil independent of the altitude above sea level. A strong correlation has been noticed between ^210^Po and ^210^Pb activities in the same parts of sclerotia, and the correlation coefficient (*r-*value) has been calculated at 0.96 for ^210^Po(ex)-^210^Pb(ex) and 0.90 for ^210^Po(in)-^210^Pb (in). However, there was no correlation between ^210^Po and ^210^Pb activities in the inner and outer parts (*r*-values ranged from 0.03 to 0.26). Moreover, the values of ^210^Po and ^210^Pb activities in the internal and external parts were significantly different (Table 2). The results suggest that the distribution of ^210^Po and ^210^Pb change as the sclerotia grow. The radionuclides amount could be an effect of their dilution in the sclerotia rather than selective bioconcentration (Szymańska and Strumińska-Parulska, 2020).

The ^210^Po/^210^Pb activity ratio values in the external part of the sclerotia were close to 1 (median and mean value of the ^210^Po/^210^Pb activity ratio were 1.12 and 1.16, respectively). In contrast, in the internal parts, the ^210^Po/^210^Pb activity ratio values were lower than 1 (median and mean value of the ^210^Po/^210^Pb activity ratio—0.80 and 0.77, respectively). A statistical analysis (Mann–Whitney *U*-test) has indicated significant differences between the values of the ^210^Po/^210^Pb activity ratio of the inner and outer parts of *W. cocos* (*p*-value 7 × 10^−6^). In the atmosphere, ^210^Po and ^210^Pb are rapidly sorbed onto aerosol particles and enter the soils due to atmospheric precipitation, and the values of ^210^Po/^210^Pb activity ratio are much lower than 1 (0.03–0.20) (Titayeva 1994; Persson 2015), whereas in biota samples, the typical ^210^Po/^210^Pb activity ratio values are close to 1 or higher (Carvalho et al., 2017). Previous research on macromycetes has confirmed the various possible mechanisms that would impact the ^210^Pb and ^210^Po presence (Vaaramaa et al., 2009; Gwynn et al., 2013). Thus, weaker uptake and lead (Pb) accumulation from the soil, atmospheric deposition, and higher selenium (an analog of polonium) accumulation influence the value of ^210^Po/^210^Pb activity ratios (Borovička and Řanda, 2007; Vaaramaa et al., 2009; Gwynn et al., 2013). However, higher lead concentrations have been observed in the saprophytic species than the mycorrhizal ones (Ángeles García et al., 2009; Strumińska-Parulska et al., 2021). Some specific geochemical and environmental conditions in the Yunnan soil, e.g., polymetallic soils, hydrology, atmospheric fallout, may also influence the ratio. Soil mushrooms, like plants, can accumulate radionuclides in the mycelium and fruiting bodies both by mycelial absorption from the ground (supported Po) and also in the fruit bodies by direct deposition from radioactive atmospheric fallout (unsupported Po) (Henricsson and Persson, 2012; Szymańska and Strumińska-Parulska, 2020). Despite this, the differences between the ^210^Po/^210^Pb activity ratio values in the examined porias’ parts could also result from radionuclides dilution during sclerotial growth rather than their selective bioconcentration directly linked to *W. cocos* morphology, unlike many other macrofungi. *Wolfiporia cocos* sclerotia typically grow for 14–24 weeks until they reach a collectible size (Kubo et al., 2006). Tyler (1982) observed that Al, As, Ca, Cd, Cu, Fe, K, Mg, Mn, Na, and Pb concentrations in soil and beech leaf litter, as well as some soil parameters (acidity, clay, and organic matter content, and HCl-extractable amount of the metal), were not the determinant for the uptake of elements by *Amanita rubescens* and *Collybia personata* mushrooms (except Rb in litter in the case of *C. personata* and of Mn in soil and *A. rubescens* and soil organic matter and Cd in this fungus), in contrast with plants. In principle, only lipophilic metal complexes or organometals can diffuse over biological membranes. In contrast, hydrophilic metal complexes, including free hydrated ions, must pass cell membranes by mechanisms other than free diffusion (Bjerregaard and Andersen, 2007). The research performed on stable Pb has shown that the impact of pH on Pb uptake is essential, and the most significant uptake capacity was found at pH = 5.5, while at pH below 3.0, uptake of lead was insignificant. Further, the amount of adsorbed Pb^2+^ per mass unit increased with the increase in initial lead ion concentration (Ezzouhri et al., 2010). The stable lead studies in some macromycetes have indicated that they produced two types of phytochelatins which played a crucial role in removing metals from soil (Damodaran et al., 2013). We might expect similar results in the case of radiolead ^210^Pb. However, this has not been investigated in the case of polonium and would need further consideration.

**Fig. 3:**
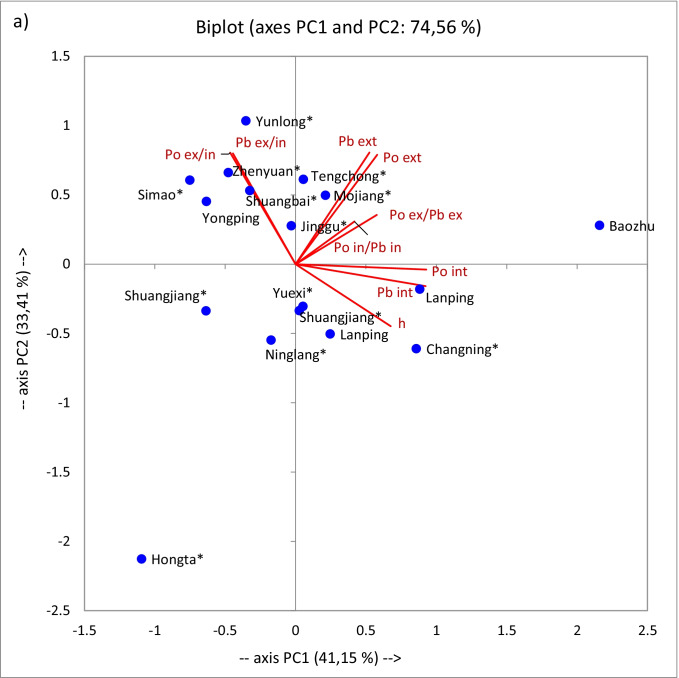
PCA biplot (no rotation) based on the activity concentrations as well as height above sea level values and score plots of the sampling sites in the space of the first and second varifactor

Principal component analyses (PCA) of datasets were carried out to characterize better the possible spatial variations in the radionuclides activity concentrations between all sampling sites from Yunnan and Anhui province; principal component analyses (PCA) of datasets were carried out (Fig. 3). The number of significant factors, total variance (%), and factor loadings are tabulated (Table 3). PCA data has revealed that 86.94% of information regarding the radionuclides compositional variability for these sites could be described by three varifactors (Table 3, Fig. 3). Because the eigenvalue of the third PC was 1.11, the third component was not examined. The factor loadings have shown that the first varifactor (PC1) explained 41.15% of the total variance and was loaded heavily towards the positively correlated variables, describing ^210^Po and ^210^Pb in the inner parts of sclerotia (loadings were 0.928 and 0.924, respectively). The second varifactor (PC2) was loaded primarily by positively correlated ^210^Po and ^210^Pb in external parts (loadings were 0.789 and 0.806, respectively) as well as the ratio of ^210^Po or ^210^Pb in the external to ^210^Po or ^210^Pb in the inner parts (loadings were 0.805 for ^210^Po and 0.799 for ^210^Pb) and accounted for 33.41% of the total variance (Fig. 3). The PCA biplot shown in Fig. 3 indicated that the ^210^Po/^210^Pb activity ratios in respective poria parts (internal or external) were the most influential variables to the PC1. In contrast, the most important contributors to the PC2 were height and ^210^Po and ^210^Pb activity concentration in the internal part. The PCA plot shows clusters of sampled locations based on their similarity (Fig. 3). There is a positive association with Tengchang and Mojiang and Bazhou—regions related to uranium and coal mines in Yunnan (Ren et al., 1999; Li et al., 2006; Wang et al., 2015a; Chen et al., 2017). Also, other studies have shown that the polymetallic soils of Wenshan (Wenshan mine, Dulong ore field–skarn type tin-zinc-polymetallic sulfide deposits), Chuxiong, Pu’er, Honghe, and Baoshan prefectures contained higher amounts of stable elements as Li, Tl, Cu, Mn, Se, Zn, Sn, Ag, and Pb as well as some radionuclides, i.e., ^40^ K and total uranium (U) (Searls, 1952; Patton Schell, 2014; Wang et al., 2015a, b; Sun et al., 2016; Falandysz et al., 2017a, b, 2020b and 2021). Thus, as a result, the *W. cocos* samples from these places also contained higher activity concentrations of ^210^Po and ^210^Pb, indirect products of ^238^U decay. Previously measured ^210^Po activity concentrations in the fruiting bodies of *Boletus bainiugan* from Yunnan turned out to be similar to those observed here for external parts of the studied sclerotia (Strumińska-Parulska et al., 2020a). Also, the ^210^Po and ^210^Pb activity concentrations in cigarette samples originating from Yunnan province were approximately 30% higher than those from Gansu province (Tokonami et al., 2008).

**Table 3:** Factor loadings after normalization (no rotation); values in bold correspond for each variable to the factor for which the squared cosine is the largest

	PC1	PC2	PC3
h	0.676	− 0.447	− 0.197
^210^Po internal	**0.928**	− 0.039	0.141
^210^Po external	0.580	**0.789**	− 0.006
^210^Pb internal	**0.924**	− 0.158	− 0.229
^210^Pb external	0.525	**0.806**	0.097
^210^Po ex/^210^Pb ex	0.577	0.355	− 0.534
^210^Po in/^210^Pb in	0.418	0.308	**0.794**
^210^Po ex/in	− 0.465	**0.805**	− 0.278
^210^Pb ex/in	− 0.445	**0.799**	− 0.030
Eigenvalue	3.703	3.007	1.114
% variance	41.149	33.409	12.383
Cumulative %	41.149	74.558	86.941

### *Effective doses from *^*210*^*Po and *^*210*^*Pb intake with analyzed parts of W. cocos*

The effective exposure doses for human consumers have been evaluated using the previously determined ^210^Po and ^210^Pb activities in the examined sclerotia (Table 4). Based on the obtained results, we have calculated the potential radiotoxicity of analyzed nuclides and the implications for food safety using the ICRP conversion coefficient recommended for ^210^Po and ^210^Pb ingestion in the case of adult members of the public (1.2 μSv·Bq^−1^ and 0.69 μSv·Bq^−1^, respectively — ICRP 2012). The effective radiation dose from food mass consumption (Sv kg^−1^ dw) was calculated as the product of the conversion coefficient (Sv Bq^−1^) and the activity concentration (Bq kg^−1^ dw). The results show that the potential consumption of dried mushrooms would result in an effective dose in the range of 0.13 to 42.4 µSv kg^−1^ dw, from ^210^Po decay, and from 0.11 to 13.6 µSv kg^−1^ dw, from ^210^Pb decay. Despite different parts of the sclerotia being used in Chinese traditional medicine and much higher activities of ^210^Po and ^210^Pb in the external parts of examined samples, one should remember that it is mostly the inner white layer (“poria,” “bai-fu-ling”) and the core (“fu-shen”) that is used (Leung and Foster, 1996). This way, the most probable, hypothetical radiotoxicity of *W. cocos* would come from the available nutrition products and reach the effective dose in the range from 0.13 to 3.43 µSv kg^−1^ dw or from 0.11 to 1.52 µSv kg^−1^ dw, in the case of ^210^Pb decay, (Table. 4). Rios (2011) recommended daily intake (dosage) of the fungus from 3 to 45 g depending on the medical problem. Thus, consumption may vary, but our calculations allow estimation of the radiation doses individually based on personal consumption. Additionally, the results obtained also allow the potential annual effective dose estimation. The recommended daily intake of the inner part consumption would result in an annual effective dose of 0.14 to 56.4 µSv a^−1^ dw from ^210^Po decay and 0.12 to 25.0 µSv a^−1^ dw from ^210^Pb decay. However, in the case of the external part consumption, it might be much higher and result in an annual effective dose of 0.88 µSv a^−1^ dw to 0.70 mSv a^−1^ dw from ^210^Po decay while of 0.58 µSv a^−1^ dw to 0.22 mSv a^−1^ dw from ^210^Pb decay. The calculated values of effective doses from ^210^Po and ^210^Pb ingestion with *W. cocos* are similar or lower (in the case of inner parts) in comparison to other studies of wild fungi from other parts of the world (Hendry et al., 2009; Vaaramaa et al., 2009; Gwynn et al., 2013; Guillén and Baeza, 2014; Bagchi and Swaroop, 2016; Strumińska-Parulska et al., 2016; 2017; 2020a; 2020b; 2021; Szymańska et al., 2018, 2019; Pang et al., 2019; Poschl and Nollet, 2019; Strumińska-Parulska and Falandysz 2020). In general, when consumed with staple foodstuffs, the analyzed sclerotia would not significantly increase the values of the effective radiation doses from ^210^Po and ^210^Pb isotopes. Therefore, the consumption of *W. cocos* sclerotia appears to be safe from a radiological protection point of view (IAEA – International Atomic Energy Agency 1996). Even the increase in popularity of *W. cocos* as a nutrition supplement does not carry radiological risk.

**Table 4:** The values of potential effective doses and measurement uncertainty from ^210^Po and ^210^Pb taken with sclerotia of *W. cocos*; *specimens from cultivation

No	Collection site	^210^Po (μSv kg^−1^ dw)		^210^Pb (μSv kg^−1^ dw)	
		Internal part	External part	Internal part	External part
1	Yuanjiang,Yuxi	0.14	n/a	0.17	n/a
2	Jianshui, Honghe	0.13	n/a	0.14	n/a
3	Tengchong, Baoshan	0.16	n/a	0.12	n/a
4	Huize, Qujing	0.17	n/a	0.18	n/a
5	Shiping, Honghe	0.15	n/a	0.11	n/a
6	Kaiyuan, Honghe	0.15	n/a	0.15	n/a
7	Octagonal town, Chuxiong	0.78	n/a	0.53	n/a
8	Xincun town, Chuxiong	0.20	n/a	0.19	n/a
9	Nanhua, Chuxiong	0.39	n/a	0.23	n/a
10	Yongping, Dali	0.20	11.9	0.17	6.63
11	Lanping, Nujiang	0.54	10.0	0.46	5.95
12	Hongta, Yuxi*	0.15	0.80	0.11	0.53
13	Changning, Baoshan*	0.79	14.0	0.72	7.08
14	Zhenyuan, Pu'er*	0.23	16.5	0.22	7.74
15	Shuangbai, Chuxiong*	0.28	13.8	0.19	7.09
16	Baozhu, Wenshan	3.43	42.4	1.52	13.6
17	Lanping, Nujiang	0.87	15.9	0.58	6.70
18	Ninglang, Lijiang*	0.25	6.16	0.19	3.18
19	Tengchong, Baoshan*	0.37	19.7	0.25	10.1
20	Shuangjiang, Lincang*	0.25	9.87	0.26	4.15
21	Yunlong, Dali*	0.24	21.0	0.16	10.5
22	Jinggu, Pu'er*	0.36	14.6	0.25	8.38
23	Shuangjiang, Lincang*	0.16	6.73	0.18	3.40
24	Mojiang, Pu'er*	0.36	18.9	0.26	7.88
25	Simao, Pu'er*	0.18	12.2	0.14	6.36
26	Yuexi, Anhui Province*	0.36	9.43	0.25	6.14

*n/a* samples not available

It should be noted that the entire quantity of ^210^Po and ^210^Pb contained in consumed sclerotia cannot be absorbed from the gastrointestinal tract. Various studies have shown that absorption of Po from foods is around 50% (ICPR 1993), about 56% (30–70%) from the consumption of caribou meat (Thomas et al., 2001), and considerably higher at 60 to 94%, (mean 76%) from the consumption of crab meat (7 volunteers; daily excretion of 10–25% in the feces and 0.2–0.3% in the urine) (Hunt and Allington, 1993). Nevertheless, the lower the activity concentration, the greater the absorption. For ^209^Po dosed orally, absorption was reported to range from 50 to 75%, with a biological half-life of 30 to 40 days (Henricsson et al., 2012). Fungal cell walls contain chitin (a fiber-like polysaccharide) that is hardly digestible by humans. As mentioned, the sclerotial chitin content in the form of beta-glucans is high, reducing the ^210^Po and ^210^Pb absorption. Still, there are no known studies as yet, to confirm this.

## Conclusions

This study presents the first results of ^210^Po and ^210^Pb occurrence in the sclerotia of the medicinal fungus *Wolfiporia cocos* from wild and cultivated sources. ^210^Po and ^210^Pb activity concentrations varied widely, and their occurrence in the sclerotia has been correlated. In the studied areas, the activity concentrations of ^210^Po and ^210^Pb in *W. cocos* samples have been independent of the altitude above sea level. Higher activities of ^210^Po and ^210^Pb have been measured in the external compared to the inner parts of the sclerotia, possibly because of radionuclide dilution during sclerotial biomass growth rather than their selective bioconcentration. At some of the study locations, the proximity of uranium and coal mines may influence higher ^210^Po and ^210^Pb activity concentrations in *W cocos*. The effective exposure doses for human consumers have been calculated for traditional Chinese medicine’s most popular fungal product. This hypothetical radiotoxicity of *W. cocos* has been evaluated at a low level. From the perspective of food safety, the consumption sclerotia of *W. cocos* seem to be safe from a radiological protection point of view. There should not be any risk connected to the occurrence of ^210^Po and ^210^Pb in its nutritional and medicinal products.

## Data Availability

The datasets used during the study are available from the corresponding author on request.
